# Impact of cone-beam computed tomography with automated feeder detection software on the survival outcome of patients with hepatocellular carcinoma during treatment with conventional transarterial chemoembolization

**DOI:** 10.1186/s12876-021-02004-z

**Published:** 2021-11-08

**Authors:** Kittipitch Bannangkoon, Keerati Hongsakul, Teeravut Tubtawee

**Affiliations:** Department of Radiology, Faculty of Medicine, Prince of Songkla University, Hat Yai, Songkhla 90110 Thailand

**Keywords:** Cone-beam computed tomography, Software tools, Survival rate, Hepatocellular carcinoma, Therapeutic chemoembolization

## Abstract

**Background:**

Inoperable hepatocellular carcinoma (HCC) is treated by conventional transarterial chemoembolization (cTACE) using cone-beam computed tomography (CBCT) or digital subtraction angiography (DSA). We compared patient survival outcomes between CBCT-cTACE with automated tumor-feeder detection (AFD) software and DSA-cTACE alone in inoperable HCC patients.

**Methods:**

We reviewed the data of 337 HCC patients treated by CBCT-cTACE or DSA-cTACE between January 2015 and December 2019. Treatment response, progression-free survival (PFS), overall survival (OS), and complications between the CBCT-cTACE and DSA-cTACE groups were compared. Univariate and multivariate logistic regression analyses examined the potential prognostic factors affecting survival after chemoembolization.

**Results:**

Tumor response rates in complete response, partial response, and stable disease at 1 month were 67%, 28%, and 4% in the CBCT-cTACE group and 22%, 48%, and 9% in the DSA-cTACE group, respectively. OS rates of patients in the CBCT-cTACE versus DSA-cTACE groups were 87% versus 54%, 44% versus 15%, and 34% versus 7% at 1, 3, and 5 years, respectively. The CBCT-cTACE group had significantly improved PFS (*p* < 0.001) and OS (*p* < 0.001). Multivariate analysis showed that CBCT with AFD software was an independent factor associated with longer OS (hazard ratio, 0.38; *p* < 0.001).

**Conclusions:**

Compared with conventional DSA, combining selective cTACE with CBCT and AFD software leads to better tumor response and prolongs OS in patients with inoperable HCC.

## Background

Conventional transarterial chemoembolization (cTACE) is the standard treatment in patients with inoperable hepatocellular carcinoma (HCC), especially in Barcelona Clinic Liver Cancer (BCLC) stage B (intermediate stage) [1–3]. It is crucial to perform selective catheterization of all tumor-feeding arteries and precisely administer chemotherapeutic drugs and iodized oil to the tumor for effective cTACE. However, inoperable stage HCC varies in terms of tumor size, number of tumor nodules, and complex multiple hepatic arterial branches that require multiple angiographic runs. Two-dimensional digital subtraction angiography (DSA) is routinely used for cTACE procedures to identify the tumor-feeding branches and for tumor targeting. However, conventional DSA has some limitations in terms of identifying small tumor nodules and their feeding-arteries. Furthermore, DSA does not accurately assess accumulation of the iodized oil inside the tumors that might affect tumor recurrence and prognostic outcome [4, 5].

Nowadays, C-arm cone-beam computed tomography (CBCT) is a valuable tool for obtaining three-dimensional images providing additional information including visualization of subtle tumor nodules, tiny hepatic feeding arteries, extrahepatic collateral arteries, and to monitor the embolized area during the cTACE procedure [6–9]. Iwazawa et al. [10] reported that CBCT in addition to DSA during cTACE can prolong survival in unresectable HCC patients.

Automated tumor-feeder detection (AFD) software is a new promising technology for the identification of intrahepatic and extrahepatic tumor-feeding arteries using CBCT data. The rates of identifying tumor-feeding arteries by AFD software were reported to be from 88 to 90% higher than using DSA guidance [7, 11, 12]. Accordingly, CBCT with AFD software has become an essential tool for performing cTACE safely and effectively in the treatment of liver tumors. To our knowledge, the efficacy of intraprocedural CBCT with AFD software assisted cTACE on survival outcome in unresectable HCC patients has not been identified in the literature. Therefore, for the first time, we retrospectively compared the survival outcome in patients who underwent CBCT using AFD software assisted cTACE and patients who underwent cTACE using DSA alone. We also evaluated the prognostic factors affecting the survival in HCC patients.

## Methods

### Ethics statement

This study complies with the standards of the Declaration of Helsinki and current ethical guidelines, and approval was obtained from the institutional ethics committee (REC No. 64-257-7-1). The requirement for informed consent for this study was waived by the Institutional Review Board, and all the data were analyzed anonymously.

### Patient population

We followed the American Association for the Study of Liver Disease criteria for the diagnosis of HCC [13]. Hepatic lesions larger than 1 cm were assessed by multiphasic contrast-enhanced computed tomography (CT) or dynamic magnetic resonance imaging (MRI). If the imaging characteristics were the typical appearance of HCC, no further diagnostic procedure was attempted.

The data of 337 HCC patients treated with selective cTACE between January 2015 and December 2019 at our hospital were collected for this study. Eligible patients for inclusion included: (1) patients aged > 18 years, (2) HCC patients with tumor size ≤ 7 cm in diameter and the number of nodules ≤ 5, (3) patients ineligible for surgical resection or transplantation, (4) patients treated with selective cTACE, and (5) patients who underwent imaging examination by either dynamic MRI or 4-phase contrast-enhanced CT scan within one month after the initial procedure. The excluded patients were those with severe impaired hepatic function (Child–Pugh class C), extrahepatic metastasis, concomitant malignancy, severe arterioportal shunt, history of spontaneous tumor rupture, presence of vascular invasion, and cotreatment with any systemic or locoregional therapies during the cTACE session.

Of the 337 HCC patients included in this study, 141 underwent cTACE using DSA alone and 196 underwent cTACE with CBCT (with or without AFD software) between January 2015 and December 2019. We assigned our patients to each technique on the basis of a discussion with interventional radiology staff. To minimize selection bias, patients treated by both techniques (i.e., first treated by cTACE with DSA alone and subsequently underwent cTACE by CBCT) were excluded from this study.

### Selective cTACE protocols

All HCC patients underwent selective cTACE using an angiographic system (Allura Clarity FD20, Philips Healthcare, Eindhoven, the Netherlands) under the supervision of two interventional radiologists with more than 8 years of experience in body interventional radiology through the transfemoral route. Celiac and superior mesenteric arteries were selected at the beginning of the procedure using a 5-Fr diagnostic catheter and a 0.035-inch J-tip guidewire. We performed selective catheterization to the tumor feeding hepatic arteries or in extrahepatic collaterals as distal as possible in each tumor lesion using a 1.98-Fr to 2.4-Fr microcatheter. After the microcatheter was inserted into the target arterial feeder, we slowly administered a mixture of iodized oil (range, 2‒16 ml) (Lipiodol, Guerbet) and doxorubicin hydrochloride (range, 5 to 50 mg) (Adriamycin, Pfizer) or mitomycin (range, 10‒20 mg) (Vesimycin, Naprod Life Sciences) under real time monitoring with DSA. We prepared the cTACE mixture using the water-in-oil technique in which the emulsion contains a chemotherapeutic agent (powder form) diluted with contrast media (Iohexol 350 mgI/ml contrast agent) and subsequently mixed with iodized oil [14]. The amount of water-in-oil-emulsion (1:2 or 1:4 ratio) was adjusted according to the total tumor size and number of nodules. Subsequently, the feeding artery was embolized using gelatin sponge particles. We completed the procedure when the tumor feeding branch was completely obstructed and tumor staining from DSA completely disappeared.

### Intraprocedural CBCT technique with AFD software

**Fig. 1:**
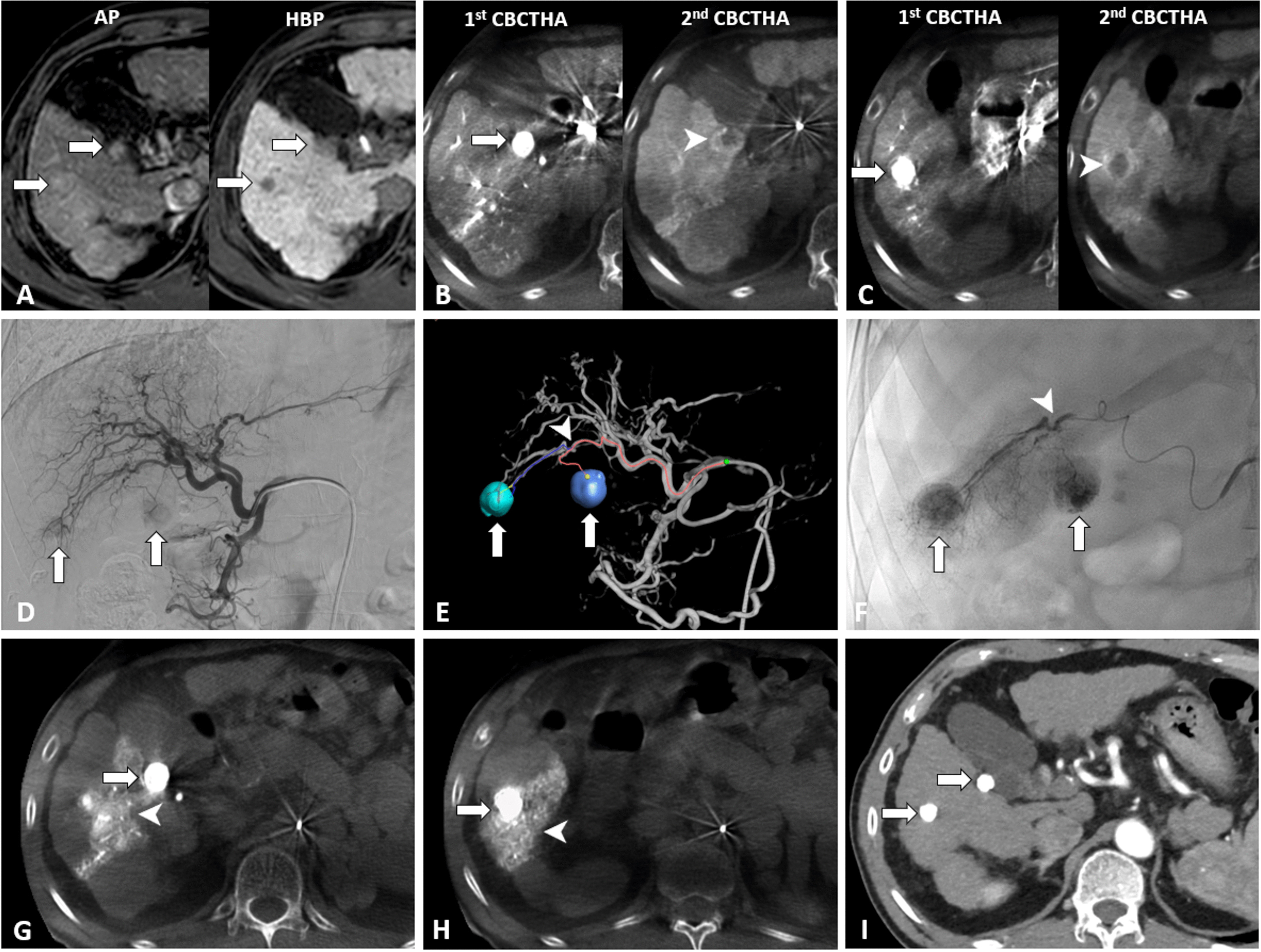
Cone-beam computed tomography (CBCT) with automated tumor-feeder detection software assisted conventional transarterial chemoembolization (cTACE). **A** Gadoxetic acid-enhanced MRI in the arterial phase in a 58-year-old male with chronic viral hepatitis-B cirrhosis showing two hyperenhancing nodules (arrows) in liver segment 5. Hepatobiliary phase imaging at 20 min after injection shows two hypointense nodules (arrows) against the background of enhancing liver parenchyma, which may indicate HCC. **B**‒**C** Dual-phase CBCT during hepatic arteriography could depict all tumors (arrows) with corona enhancement (arrowheads). **D** Common hepatic arteriogram showed two faint tumor stains (arrows). However, the tumor-feeders were unclear. **E** Automated tumor-feeder detection software identified the feeders (arrowhead) of each tumor (arrows). **F** Tumor-feeder (arrowhead) was selectively embolized during cTACE. Complete tumor staining (arrows) was demonstrated from spot image on digital subtraction angiography. **G**‒**H** CBCT immediately after cTACE showed dense iodized oil accumulation in all tumors (arrows) with a sufficient safety margin (arrowheads). **I** Enhanced CT performed 1 year after selective cTACE showed dense iodized oil accumulation in all tumors without tumor recurrence

The CBCT data were acquired using an Allura Clarity FD20 which enabled CBCT acquisition and volumetric image reconstruction. A total of 242 projection images (60 frames/second) with X-ray parameters of 121 kV and 200‒300 mAs were obtained with the motorized C-arm covering a 220° clockwise rotation of the flat panel detectors. The patients were instructed to be at end-expiratory apnea during the CBCT scanning. All CBCT images of 5 mm thickness were interpreted using a workstation (Philips Healthcare, Eindhoven, the Netherlands). We performed CBCT with AFD software assisted cTACE in three steps (Fig. 1).

#### Step 1: Identification of tumor nodules

We placed the catheter at the proper or common hepatic artery and performed CBCT during hepatic arteriography (CBCTHA). A total of 24 ml of non-diluted contrast media (Omnipaque 350 mgI/ml, Bayer, Bangkok, Thailand) was injected via a 5-Fr diagnostic catheter at the rate of 2 ml/second. The first phase of CBCTHA started after contrast material injection for 7 s and the second scan was achieved 30 s after finishing the first scan. The patients held their breath twice during the gantry rotation. All hepatic lesions with corona enhancement, which represented venous drainage through hypervascular HCC nodules [15] on the second scan of CBCTHA were targeted for treatment with cTACE.

#### Step 2: Detection of tumor feeding arteries

The automated tumor-feeder software (EmboGuide, Philips Healthcare, Best, the Netherlands) used the data from the first scan of CBCTHA to identify the tumor feeders in about 2 min at the workstation. We used AFD software to detect segmental tumor feeders in case they were not clearly seen on conventional DSA images. After the feeder branches were identified, embolization was performed without obtaining further DSA images.

#### Step 3: Evaluation of iodized oil accumulation

We performed plain CBCT without contrast media injection just after the selective cTACE procedure to monitor the distribution of iodized oil. The goal of chemoembolization is to completely embolize the entire target tumor with or without an adequate safety margin. If incomplete tumor stains occurred, further chemoembolization was performed.

### Analysis of the prognostic factors for overall survival

Univariate and multivariate analyses were used to define the significant independent factors affecting overall survival (OS). Fourteen clinical factors were analyzed: age, gender, alcohol drinking, hepatitis B or C virus carrier, Child–Pugh class, albumin-bilirubin (ALBI) score, total bilirubin, albumin, international normalized ratio (INR), platelet count, serum alpha-fetoprotein (AFP) level, ascites, and BCLC staging. Also analyzed were four tumor factors: size of largest tumor, number of tumor nodules, lobar involvement, and the up-to-7 criteria plus two procedure-related factors: complete remission at 1 month and use of CBCT-assisted cTACE.

### Outcome and treatment evaluation

All eligible patients were followed up after selective cTACE with a detailed clinical examination, blood chemistries, and an imaging study using 4-phase contrast-enhanced CT scan or dynamic MRI within one month after the initial procedure. If no definite evidence of residual or recurrent tumor presented, imaging investigation was performed subsequently at 3-month intervals. We used the modified Response Evaluation Criteria in Solid Tumors (mRECIST) to assess HCC after treatment [16]. Assessment of tumor response was reviewed independently by two radiologists with expertise in liver imaging (TT: > 10 years; KB: 8 years) to minimize variability in this critical instance [17]. In cases of discordance, the final decisions were achieved by consensus. The decision to repeat the cTACE procedure was based on tumor response by mRECIST, BCLC-staging of the disease, and the patient’s tolerance.

### Complications

cTACE-related complications were classified as major or minor following the standard Society of Interventional Radiology (SIR) guidelines [18]. Major complications of cTACE were defined as admission to a hospital for therapy, an unplanned increase in the level of care, prolonged hospitalization, permanent adverse sequelae, or death after the procedures by SIR guidelines [18].

### Data and statistical analysis

All data analyses were performed using R software (version 4.1.0). Numerical data are given descriptively using the central tendency (mean, median, and mode) and a measure of dispersion (standard deviation and range). We used chi-square or t-test to compare baseline characteristics between the HCC patients who underwent cTACE with CBCT assistance and patients who received cTACE with DSA. Local progression-free survival (PFS) was calculated from the date of selective cTACE to the last date of local tumor progression or the date of patient death. Local tumor progression was diagnosed when an arterial enhancing lesion was depicted in or adjacent to the treated tumor on follow-up imaging. OS was calculated from the date of selective cTACE to the date of patient death. Patient status at the end of the study (December 31, 2020) was defined as alive or dead using data from the Thailand civil registration database. The local PFS and OS rates for the two groups of HCC patients were compared using the Kaplan–Meier estimator. The probability of OS for the two groups was estimated using the Cox’s proportional hazards regression model. The OS rates at 1, 3, and 5 years were calculated and compared for each group.

Baseline clinical characteristics, tumor appearances, and procedure-related factors affecting survival were initially assessed by univariate analysis. Subsequently, all prognostic factors having *p* values ≤ 0.2 from the univariate analysis were entered into the initial multivariate Cox's proportional hazards regression. The model was refined by sequentially removing non-significant variables. *p* Values < 0.05 were considered statistically significant.

## Results

### Patient characteristics

A total of 337 HCC patients were treated with selective cTACE as the first treatment for HCC. One hundred and ninety-six patients were treated by cTACE with CBCT assistance (CBCT-cTACE group) and 141 patients were treated by cTACE with DSA alone (DSA-cTACE group). One hundred and fifty-one patients were categorized into BCLC-B stage and the rest were classified into BCLC stage 0 and stage A within the Milan criteria. Surgery or ablation was avoided in 186 patients in our study for these reasons: not eligible for surgery due to unfavorable remnant liver function, location of the tumor near a blood vessel or another organ, patient intention, inadequate visualization during ultrasonography, presence of ascites, advanced obesity, and severe abnormal coagulopathy.

Subsequently, baseline characteristics were compared between HCC patients in the CBCT-cTACE group and patients in the DSA-cTACE group (Table 1). There were no significant differences between the two groups in terms of age, gender, alcohol drinking, hepatitis B virus (HBV) positivity, hepatitis C virus (HCV) positivity, Child–Pugh class, ALBI score, total bilirubin level, albumin level, INR level, platelet count, serum AFP level, presence of ascites, size of the largest tumor, tumor number, lobar involvement, up-to-7 criteria, and BCLC-staging. However, compared with HCC patients in the DSA-cTACE group, the CBCT-cTACE group had a considerably lower proportion of BCLC-B HCC patients (*p* = 0.130), and trends to have higher serum albumin (*p* = 0.064) and INR (*p* = 0.052) levels.

**Table 1:** Baseline characteristics of HCC patients treated by cTACE with CBCT assistance (CBCT-cTACE) and patients treated by cTACE with DSA alone (DSA-cTACE)

Prognostic factors	CBCT-cTACE n = 196	DSA-cTACE n = 141	*p* value
Age, years, mean (range)	62.3 (21‒91)	63.3 (37‒90)	0.407
Gender			
Male	140 (71)	94 (67)	0.414
Female	56 (29)	47 (33)	
Alcohol drinking			
No	164 (84)	119 (84)	0.978
Yes	32 (16)	22 (16)	
Hepatitis B virus carrier			
No	99 (51)	76 (54)	0.614
Yes	97 (49)	65 (46)	
Hepatitis C virus carrier			
No	142 (72)	105 (75)	0.773
Yes	54 (28)	36 (25)	
Child–Pugh Class			
A (5‒6)	144 (73)	96 (68)	0.283
B (7)	37 (19)	27 (19)	
B (8‒9)	15 (8)	18 (13)	
ALBI score			
1	53 (27)	35 (25)	0.780
2	131 (67)	95 (67)	
3	12 (6)	11 (8)	
Total bilirubin, (mg/dl), median (range)	0.87 (0.18‒2.87)	0.98 (0.20‒2.95)	0.154
Albumin, (ng/ml), median (range)	3.6 (2.2‒4.7)	3.4 (1.8‒4.8)	0.064
INR, median (range)	1.25 (1.0‒2.2)	1.22 (0.9‒2.0)	0.052
Platelet count, × 10^3^/mm^3^, median (range)	99 (30‒435)	106 (35‒318)	0.853
Serum AFP level (ng/ml)			
< 20	96 (49)	56 (40)	0.241
20‒200	57 (29)	48 (34)	
> 200	43 (22)	37 (26)	
Ascites			
No	173 (88)	117 (83)	0.222
Yes	23 (12)	24 (17)	
Size of largest tumor, mm, median (range)	3.1 (1.1‒7.0)	3.2 (1.1‒7.0)	0.157
Tumor number			
1	108 (55)	72 (51)	0.179
2–3	64 (33)	58 (41)	
4–5	24 (12)	11 (8)	
Site of tumors			
Unilobar	165 (84)	118 (84)	1.000
Bilobar	31 (16)	23 (16)	
Up-to-7 criteria			
Within	158 (81)	109 (77)	0.547
Beyond	38 (19)	32 (23)	
BCLC-staging			
Stage A	115 (59)	71 (50)	0.130
Stage B	81 (41)	70 (50)	

Data are presented as number (%) unless otherwise indicated

*CBCT-cTACE* cone-beam computed tomography assisted conventional transarterial chemoembolization, *DSA-cTACE* digital subtraction angiography assisted conventional transarterial chemoembolization, *ALBI* albumin-bilirubin, *INR* international normalized ratio, *AFP* alpha-fetoprotein, *BCLC* Barcelona Clinic Liver Cancer

### cTACE procedures

A total of 628 HCC nodules in 337 patients underwent selective cTACE. The mean size of the largest tumor was 34.2 ± 15.1 mm (range, 11‒70 mm) and the mean number of treated tumor nodules per cTACE session was 1.9 ± 1.2 nodules (range, 1‒5). Most of the HCC patients had tumor nodules within the up-to-7 criteria (79.2%) and were characterized as unilobar involvement (84.0%).

Procedural details and tumor response between HCC patients in the CBCT-cTACE group and patients in the DSA-cTACE group are summarized in Table 2. In the CBCT-cTACE group, we performed selective cTACE in 196 patients in 311 hepatic segmental areas. In the DSA-cTACE group, we performed selective cTACE in 141 patients in 213 hepatic segmental areas. The mean dose of iodized oil in one session of cTACE was not significantly different between the two groups (*p* = 0.068). The mean dose of doxorubicin in one session of cTACE was 21.6 mg (range, 5‒50 mg) in the CBCT-cTACE group and 33.6 mg (range, 5‒50 mg) in the DSA-cTACE group. The mean dose of doxorubicin in the DSA-cTACE group was significantly higher than the CBCT-group (*p* < 0.001). The mean fluoroscopic time in one session of cTACE in the CBCT-group was significantly longer than in the DSA-cTACE group (16.3 vs. 12.4 min, *p* < 0.001). However, the mean number of cine acquisitions for HCC patients in the CBCT group were significantly lower than the DSA group (4.6 vs. 5.1, *p* = 0.017).

**Table 2:** Procedural details and tumor responses of the HCC patients treated by cTACE with CBCT assistance (CBCT-cTACE) and patients treated by cTACE with DSA alone (DSA-cTACE)

	CBCT-cTACE (n = 196)	DSA-cTACE (n = 141)	*p* value
Lipiodol, ml, mean (range)	7.5 ± 3.4 (2‒16)	7.8 ± 3.3 (2‒16)	0.068
Doxorubicin dose, mg, mean (range)	21.6 ± 10.6 (5‒50)	33.6 ± 17.1 (5‒50)	< 0.001*
Number of treated hepatic segments			
1	107 (54)	76 (54)	0.150
2	68 (35)	59 (42)	
3	16 (8)	5 (3)	
4	5 (3)	1 (1)	
Number of cine acquisitions, mean (range)	4.6 (1‒14)	5.1 (2‒17)	0.017*
Number of CBCT acquisitions, mean (range)	3.4 (1‒11)		
Fluoroscopic time, mins, mean (range)	16.3 (4.1‒60.4)	12.4 (3.3‒54.4)	< 0.001*
Post embolization syndrome (%)			
Yes	19 (10)	15 (11)	0.920
No	177 (90)	126 (89)	
Tumor response^a^			
CR	131 (67)	31 (22)	< 0.001*
PR	55 (28)	67 (48)	
SD	7 (4)	13 (9)	
PD	3 (1)	30 (21)	
Additional cTACE, times, mean (range)	2.7 (1‒10)	3.0 (1‒14)	0.400

Data are presented as number (%) unless otherwise indicated

*CBCT-cTACE* cone-beam computed tomography assisted conventional transarterial chemoembolization, *DSA-cTACE* digital subtraction angiography assisted conventional transarterial chemoembolization, *CR* complete response, PR partial response, *SD* stable disease, *PD* progressive disease

^a^Tumor response defined by modified Response Evaluation Criteria in Solid Tumors Criteria (mRECIST)  ﻿

*Likelihood ratio test﻿

The mean number of CBCT acquisitions for the HCC patients in the CBCT-cTACE group was 3.4 times (range, 1‒11). In our study, CBCT was used to identify tumor nodules, detect tumor feeding arteries with AFD software, and evaluate iodized oil accumulation. First, of the 196 HCC patients in the CBCT-cTACE group, the CBCT showed additional HCC nodules that were not evident on CT, MRI, or angiography in 34 patients (17.4%). A total of 48 new HCC nodules were detected on CBCT images (1 new nodule in 23 patients, 2 new nodules in 8 patients, and 3 new nodules in 3 patients). The mean size of the newly detected HCC nodules was 10 ± 3.1 mm (range, 5‒21 mm). All newly found HCC nodules were subsequently treated with selective cTACE. Second, we used the AFD software to detect all segmental tumor-feeding arteries in 139 of the 196 patients (70.9%) when the feeding arteries were not clearly seen on conventional DSA images. The tumor feeding arteries were correctly detected by the AFD software in 133 of 139 patients (95.7%). The reasons for failure to detect the feeding arteries in six patients were: motion artifacts (4 patients), hypovascular tumor nodule (1 patient), and very small caliber feeder vessel to the tumor (1 patient). Third, we monitored the distribution of the iodized oil immediately after the selective cTACE procedure. If iodized oil deposition in the tumors was incomplete, further selective chemoembolization was performed.

Post-treatment assessments were evaluated by 4-phase contrast-enhanced CT scan or dynamic MRI 4 weeks after initial selective cTACE. Tumor response rates by mRECIST in the CBCT-cTACE group with complete response (CR), partial response (PR), stable disease (SD), and progressive disease (PD) were 67% (131 patients), 28% (55 patients), 4% (7 patients), and 1% (3 patients), respectively. In the DSA-cTACE group, the CR, PR, SD, and PD response rates were 22% (31 patients), 48% (67 patients), 9% (13 patients), and 21% (30 patients), respectively. The response rates after selective cTACE were significantly better in the CBCT-cTACE group than in the DSA-cTACE group (*p* < 0.001). The number of additional cTACE procedures was not significantly different between the two groups (*p* = 0.400).

### Complications

All cTACE-related complications were classified as minor. The most common procedure-related complication was post-embolization syndrome in 34 patients without requiring extended stay or re-admission. Post-embolization syndrome was not significantly different between the CBCT-cTACE and DSA-cTACE groups (*p* = 0.920). Groin hematoma was found in eight patients without any treatment. Minimal iodized oil stains in the gallbladder wall due to non-target embolization without symptomatic cholecystitis was found in two patients in the DSA-cTACE group without further treatment. Biloma occurred in one patient and was followed up without any treatment due to no symptoms. The remaining 221 patients were alive at the time of analysis.

### Progression-free survival

The mean follow-up time for the HCC patients who underwent selective cTACE was 20.0 ± 15.3 months (range, 0.4‒71.1). The overall recurrence rate was about 96% (136/141 patients) in the DSA-cTACE group and about 79% (155/196 patients) in the CBCT-cTACE group. The median tumor PFS periods for HCC patients in the DSA-cTACE and CBCT-cTACE groups were 33 days (95% CI 32‒35 days) and 467 days (95% CI 262‒848 days), respectively. The median PFS rates for HCC patients in the CBCT-cTACE group were significantly higher than in the DSA-cTACE groups at 1 year (52% vs. 10%), 3 years (38% vs. 6%), and 5 years (38% vs. 4%) by log-rank test (*p* < 0.001).

### Overall survival of patients in the CBCT-cTACE and DSA-cTACE groups

**Fig. 2:**
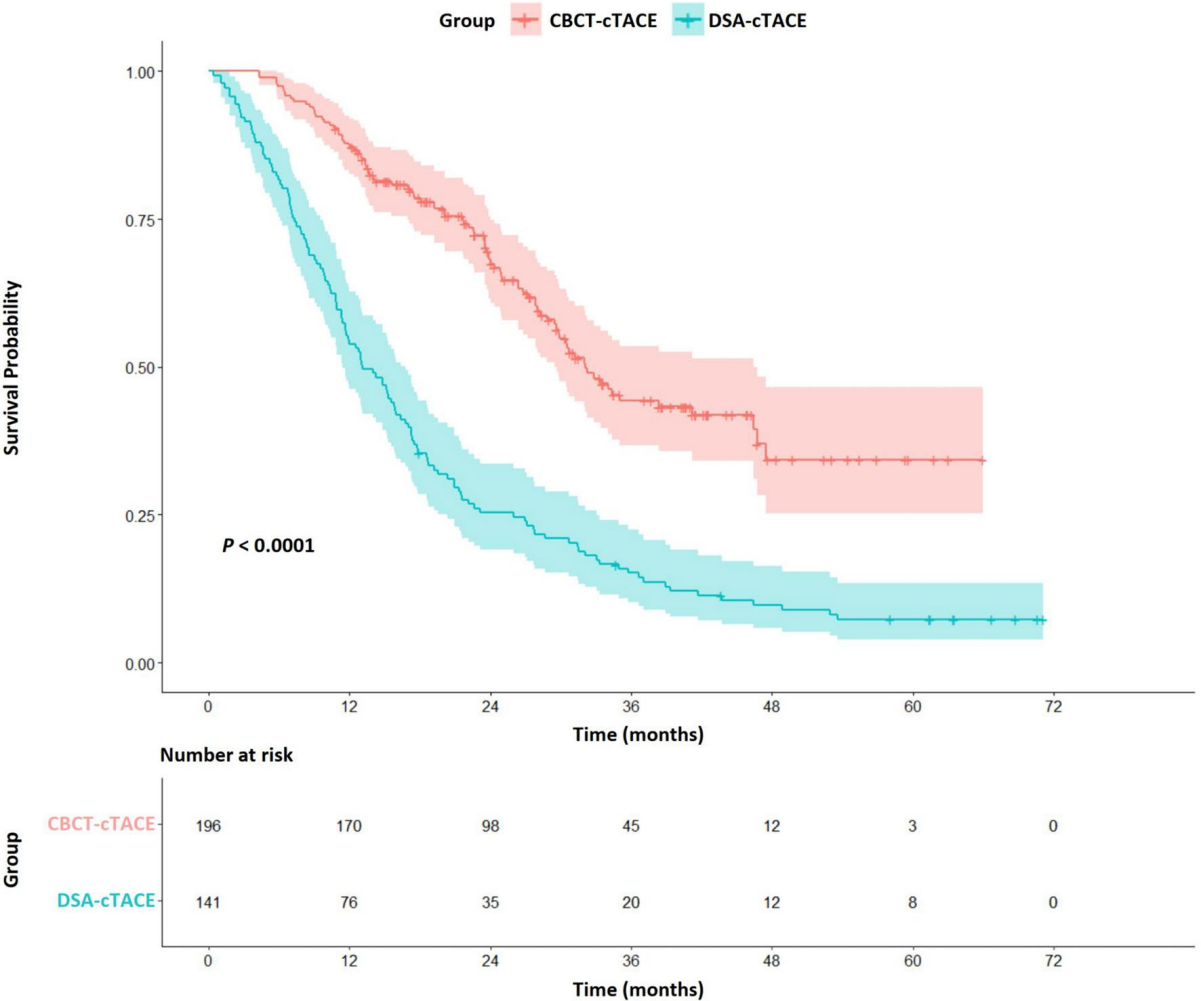
Cumulative overall survival of HCC treated with selective conventional transarterial chemoembolization (cTACE) with cone-beam computed tomography (CBCT) and automated tumor-feeder detection software assistance (CBCT-cTACE group) or with digital subtraction angiography (DSA-cTACE group)

The median OS time of the entire cohort was 23.9 months (95% CI 21.5‒27.9 months). The median OS times in the DSA-cTACE and CBCT-cTACE groups were 13.1 months (95% CI 11.4‒16.8 months) and 32.1 months (95% CI 29.5‒46.4 months), respectively. The cumulative survival rates in the CBCT-cTACE group were significantly higher than in the DSA-cTACE group at 1 year (87% vs. 54%), 3 years (44% vs. 15%), and 5 years (34% vs. 7%) by log-rank test (*p* < 0.001) (Fig. 2). The mortality rate was 91.5% (129/141 patients) in the DSA-cTACE group and 46.9% (92/196 patients) in the CBCT-cTACE group.

### Prognostic factors of overall survival

Among the 20 prognostic factors affecting OS, univariate analysis revealed that Child–Pugh class A (*p* < 0.001), ALBI score 1 or 2 (*p* < 0.001), total bilirubin level ≤ 1.0 mg/dl (*p* < 0.001), albumin > 3.5 g/dl (*p* < 0.001), INR ≤ 1.2 (*p* < 0.001), serum AFP level ≤ 200 ng/ml (*p* = 0.041), no presence of ascites (*p* = 0.005), and BCLC staging 0 or A (*p* < 0.001) were the significant clinical factors. Considering the tumor factors, size of largest tumor ≤ 3 cm in diameter and single HCC nodule were significant tumor factors on univariate analysis (*p* = 0.012 and *p* = 0.011). In addition, use of CBCT assisted cTACE and presence of CR at 1 month after cTACE were significant procedure-related factors (*p* < 0.001 and *p* < 0.001) (Table 3). Multivariate analysis for the potential prognostic factors affecting OS showed that ALBI score 1 or 2 (hazard ratio [HR]: 0.48; *p* < 0.001), serum AFP level ≤ 200 ng/ml (HR: 0.63; *p* = 0.003), presence of CR at 1 month after cTACE (HR: 0.55; *p* < 0.001), and use of CBCT assisted cTACE (HR: 0.38; *p* < 0.001) were the only four independently significant prognostic factors associated with longer OS (Table 3).

**Table 3:** Results of univariate and multivariate analysis on potential prognostic factors affecting survival after chemoembolization

Prognostic factors	Number of cases	Univariate analysis		Multivariate analysis	
		HR (95% CI)	*p* value	HR (95% CI)	*p* value
Age (≤ 65/ > 65 years)	200/137	0.91 (0.70‒1.20)	0.512		
Gender (male/female)	234/103	0.88 (0.66‒1.18)	0.392		
Alcohol drinking (no/yes)	283/54	0.88 (0.61‒1.25)	0.470		
Hepatitis B virus carrier (no/yes)	175/162	0.99 (0.76‒1.29)	0.956		
Hepatitis C virus carrier (no/yes)	247/90	1.01 (0.75‒1.35)	0.963		
Child–Pugh score (A/B)	240/97	0.56 (0.43‒0.74)	< 0.001		
ALBI score (1, 2, and 3)	88/249	0.49 (0.35‒0.69)	< 0.001	0.48 (0.34‒0.68)	< 0.001*
Total Bilirubin (≤ 1.0/ > 1.0 mg/dl)	194/143	0.59 (0.45‒0.77)	< 0.001		
Albumin (> 3.5/ ≤ 3.5 g/dl)	159/178	0.47 (0.36‒0.62)	< 0.001		
INR (≤ 1.2/ > 1.2)	138/199	0.61 (0.46‒0.80)	< 0.001		
Platelet (> 10^5^/ ≤ 10^5^ mm^3^)	170/167	0.81 (0.62‒1.05)	0.113		
Serum AFP level (≤ 200/ > 200 ng/ml)	257/80	0.73 (0.54‒0.99)	0.041	0.63 (0.46‒0.86)	0.003*
Ascites (no/yes)	290/47	0.60 (0.42‒0.86)	0.005		
BCLC-staging (0‒A/B)	186/151	0.64 (0.49‒0.83)	< 0.001		
Size of largest tumor (≤ 3/ > 3 cm)	151/186	0.71 (0.54‒0.93)	0.012		
Single tumor (yes/no)	180/157	0.71 (0.54‒0.92)	0.011		
Unilobar involvement (yes/no)	283/54	0.82 (0.58‒1.16)	0.262		
Up-to-7 criteria (within/beyond)	267/70	0.88 (0.64‒1.22)	0.447		
CR at 1 month after cTACE (yes/no)	162/175	0.34 (0.26‒0.46)	< 0.001	0.55 (0.40‒0.75)	< 0.001*
CBCT with AFD software assisted cTACE (yes/no)	196/141	0.34 (0.26‒0.44)	< 0.001	0.38 (0.28‒0.51)	< 0.001*

*HR* hazard ratio, 95% *CI* 95% confidence interval, *ALBI* albumin-bilirubin, *INR* international normalized ratio, *AFP* alpha-fetoprotein, *BCLC* Barcelona Clinic Liver Cancer, *CR* complete response, *CBCT* cone-beam computed tomography, c*TACE* conventional transarterial chemoembolization

*Likelihood ratio test

## Discussion

In our study, the additional information provided by CBCT resulted in a change in treatments, including visualization of angiographically occult HCC nodules in 34 patients (17.4%) in the CBCT-cTACE group, that were not demonstrated on CT, MRI, or angiography. All 48 small HCC nodules (mean diameter, 10.0 ± 3.1 mm) that could not be detected on other images could be depicted by dual-phase CBCTHA. These findings suggested that the sensitivity of HCC detection increases using CBCT assistance, as reported in previous studies [8, 19, 20]. Additionally, we used the corona enhancement on second phase CBCTHA images to distinguish between HCC nodules and hypervascular pseudolesions [15]. Corona enhancement represents venous drainage through hypervascular HCC and is detected in 89% of HCC nodules [21]. On the contrary, pseudolesions, such as arterioportal shunts, do not demonstrate corona enhancement [20]. Subsequently, all HCC lesions with corona enhancement on the second scan of CBCTHA were targeted for selective cTACE.

The volumetric information from CBCT provides a rotational three-dimensional map of the hepatic arterial anatomy and detection of tumor-feeding branches for selective cTACE. Based on our study, tumor feeders were correctly detected by the AFD software in 95.7% (133/139 patients), which was similar to previous studies [6, 22, 23] that reported the detectability rate of the vessel tracking software was about 81% to 93%. When a tumor-feeding branch was identified by AFD software, we performed selective cTACE of that branch without performing additional DSA runs. These results reduced the number of cine acquisitions in the CBCT group compared to the DSA group (4.6 vs. 5.1; *p* = 0.017). A previous study [24] demonstrated that the feeder-vessel detection software also reduced the overall procedural time in cTACE. However, mean fluoroscopic time in the CBCT-cTACE group was significantly longer than in the DSA-cTACE group (16.3 vs. 12.4 min, *p* < 0.001). This occurred probably because the patients in the CBCT-cTACE group had small and subtle tumor-feeding branches that required more fluoroscopic time for selective catheterization. Some limitations of CBCT images are due to motion artifacts caused by inadequate breath-holding. We found that motion artifacts causing failure to detect feeding arteries using AFD software was 2.9% in the CBCT-cTACE group, which was similar to the 3% of patients in a previous report [25]. We can reduce these motion errors by training the patients to hold their breath before the procedures and performing the dual-phase CBCTHA in a propeller-fashion (from the head-end of the table) that requires a scan time of 5 s.

The goal of selective cTACE is complete embolization of the target HCC nodule with an adequate safety margin that leads to a good therapeutic outcome and lower rate of tumor recurrence [9]. CBCT during selective cTACE can verify the accumulation of iodized oil and guide the operator on the endpoint of chemoembolization. If, immediately after selective cTACE, the CBCT shows a non-enhancing portion within the hypervascular HCC nodule, it indicates one of three possibilities: (1) incomplete embolization of the feeding artery, (2) some small feeding arteries were missed, or (3) the presence of an extrahepatic collateral artery supplying the tumor.

In our study, the tumor response rate after selective cTACE was significantly better in the CBCT-cTACE group than in the DSA-cTACE group (*p* < 0.001), even though the mean dose of chemotherapeutic drugs was significantly lower in the CBCT-cTACE group. These findings suggested that modification of the procedure with cTACE guidance software can decrease local tumor recurrence and improve the tumor response, which were similar to a previous report [26] while reducing the amount of chemoembolic agents. Although post-embolization syndrome was not significantly different between the CBCT-cTACE and DSA-cTACE groups in our study, iodized oil stains in the gallbladder were found only in two patients in the DSA-cTACE group. It was reported that non-target embolization after cTACE through the cystic artery can cause serious complications, such as gallbladder infarct, that require surgery [27].

Our study determined that use of CBCT assisted cTACE is an independently significant prognostic factor associated with longer OS (HR: 0.38; *p* < 0.001), which was similar to a previous study by Iwazawa et al. [10]. However, there are some differences in terms of methodology. In a previous study [10], the tumor-feeding branch that supplied the target tumor was clarified manually from CBCT images and confirmed by testing with contrast injection to determine whether the entire tumor was totally enhanced. In contrast, we routinely used AFD software to detect segmental tumor-feeding arteries when they were not clearly seen on conventional DSA images. When the feeder branches were labeled from the cTACE guidance software, the branches were embolized without performing additional DSA images. In the Iwazawa study [10], the median OS time in the CBCT-cTACE group without AFD software was 27.2 months (95% CI 0.7‒49.3 months), which was shorter compared to the CBCT-cTACE group with AFD software guidance in our study (32.1 months, 95% CI 29.5‒46.4 months). Therefore, CBCT with cTACE guidance software contributed to longer patient survival compared to HCC patients who underwent cTACE with DSA guidance and was possibly higher than the patients treated with CBCT aided cTACE but without AFD software assistance.

Overall survival in HCC patients can be influenced by other clinical factors, such as hepatitis viral infection or diabetes [28]. Treating the underlying etiology and concomitant liver disease should lead to better survival outcome. Furthermore, recent advances in systemic treatment for HCC, which include second line agents such as tyrosine kinase inhibitors or immunotherapy, appear to accelerate earlier decisions on whether to repeat cTACE or switch to systemic therapy [29, 30]. The concept of switching to systemic agents might be of little benefit once liver function deterioration develops after repeated cTACE. Therefore, performing curative cTACE using CBCT technology is crucial when the tumor is localized and controllable.

In comparison with cTACE, TACE with drug-eluting beads (DEB-TACE) enables higher concentrations of drugs in the distal tumor vessels resulting in more localized chemotherapeutic delivery, but lower systemic concentrations [31]. However, the microspheres in DEB-TACE cannot embolize tumor drainage, which is contrary to cTACE [32]. Therefore, reversed flow from the surrounding portal venules into the peripheral tumor portion may cause tumor tissue to survive. Randomized controlled trials conducted in Europe showed no significant differences in tumor response and survival between the DEB-TACE and cTACE groups [33–35]. On the other hand, the latest randomized controlled trial conducted in Japan demonstrated significant differences in CR rates between the cTACE and DEB-TACE groups [36]. Therefore, a discrepancy of outcomes of cTACE exists between Europe and Japan, which was possibly due to different devices and techniques. Selective cTACE uses smaller microcatheters (1.7-F to 2.0-F tip) under intraprocedural monitoring by CBCT, which is the standard technique in Japan and in our institution in Thailand. Moreover, there is a debate about the long-term cost-effectiveness of DEB-TACE compared to cTACE [37], which can be assessed in future studies.

Our study has some limitations. First, this study was retrospective in nature conducted at a single center. Second, we did not evaluate the types of microcatheters, skill of the cTACE operators or level of selective catheterization that may influence the interpretation of the survival outcome. Third, histological confirmation of HCC was not obtained. All study lesions were diagnosed as HCC based on imaging findings and elevated serum levels of tumor markers. Finally, our sample size was relatively small, which limited the number of patients available for a subgroup analysis.

In conclusion, the CBCT technology with AFD software can provide additional information during selective cTACE including identification of HCC nodules, detection of tumor feeding arteries as a 3D navigation tool, and evaluation of iodized oil accumulation, which affects the treatment response and prolongs survival in patients with inoperable HCC. The combination of selective cTACE with the capabilities of CBCT and cTACE guidance software in daily clinical practice may improve the efficacy and prognostic outcome of HCC patients.

## Data Availability

All analyzed data are included in this published article. The original data are available upon reasonable request to the corresponding author.
